# Global Temporal Patterns of Age Group and Sex Distributions of COVID-19

**DOI:** 10.3390/idr13020054

**Published:** 2021-06-21

**Authors:** Russell Leong, Tin-Suet Joan Lee, Zejia Chen, Chelsea Zhang, Jianping Xu

**Affiliations:** 1 Faculty of Health Sciences, McMaster University, Hamilton, ON L8S 4K1, Canada; leongr2@mcmaster.ca (R.L.); leet53@mcmaster.ca (T.-S.J.L.); chenz262@mcmaster.ca (Z.C.); zhanc99@mcmaster.ca (C.Z.); 2 Department of Biology and Institute of Infectious Diseases Research, McMaster University, Hamilton, ON L8S 4K1, Canada

**Keywords:** national data, COVID-19 cases, COVID-19 hospitalizations, COVID-19 deaths, case fatality rates, weekly changes

## Abstract

Since the beginning of 2020, COVID-19 has been the biggest public health crisis in the world. To help develop appropriate public health measures and deploy corresponding resources, many governments have been actively tracking COVID-19 in real time within their jurisdictions. However, one of the key unresolved issues is whether COVID-19 was distributed differently among different age groups and between the two sexes in the ongoing pandemic. The objectives of this study were to use publicly available data to investigate the relative distributions of COVID-19 cases, hospitalizations, and deaths among age groups and between the sexes throughout 2020; and to analyze temporal changes in the relative frequencies of COVID-19 for each age group and each sex. Fifteen countries reported age group and/or sex data of patients with COVID-19. Our analyses revealed that different age groups and sexes were distributed differently in COVID-19 cases, hospitalizations, and deaths. However, there were differences among countries in both their age group and sex distributions. Though there was no consistent temporal change across all countries for any age group or either sex in COVID-19 cases, hospitalizations, and deaths, several countries showed statistically significant patterns. We discuss the potential mechanisms for these observations, the limitations of this study, and the implications of our results on the management of this ongoing pandemic.

## 1. Introduction

As of 1 June 2021, the coronavirus disease of 2019 (COVID-19) has infected 171,916,673 people and led to 3,575,505 deaths [1,2]. The virus responsible for this disease—severe acute respiratory syndrome coronavirus 2 (SARS-CoV-2)—belongs to the beta-coronavirus family and has notable similarities to a previously reported coronavirus, SARS-CoV [3]. First detected in late December of 2019, COVID-19 has since been declared a global pandemic on 11 March 2020, by the World Health Organization [4]. While a large body of knowledge about COVID-19 has been gained about the mode of transmission, viral genome evolution, pathogenesis, and treatments and prevention, significant gaps remain in our understanding of the temporal patterns of age group and sex distributions of COVID-19. For example, do the relative frequencies of COVID-19 infection for people in different age groups change over time? If so, which age group(s) shows increased frequencies over time as the pandemic progresses? Are infections and/or deaths distributed across age groups similarly among countries? Do case hospitalization and case fatality rates change over time among age groups? Similarly, do the two sexes show similar patterns of COVID-19 infections, hospitalizations, and mortality? Answers to these and other questions will have important implications for the further development of public health policies to control and prevent the spread of COVID-19. The objective of this report is to investigate the differences and patterns of COVID-19 among age groups and between the two sexes at a global level. This was achieved through analysis of national age- and sex-stratified data available in the public domain.

Many countries have been tracking the temporal changes of COVID-19 cases and deaths. However, countries have differed widely in their reporting. While some countries have noted the sex and age distributions of their daily/weekly COVID-19 cases and deaths, most have not. Similarly, while a large number of epidemiological analyses for COVID-19 have been conducted, most published reports have come from specific hospitals, individual jurisdictions (such as a city or a province), or individual countries [5,6,7,8]. To date, there has been no comparative analysis of the temporal patterns of COVID-19 distributions involving multiple countries spanning the pandemic between 1 January and 31 December 2020.

The published reports so far have indicated significant differences among people of certain age groups in the severity of disease symptoms due to COVID-19 infection. In most cases, COVID-19 leads to mild or asymptomatic outcomes for younger age groups (defined as below age 45) [9]. However, COVID-19 can result in severe illness and death in certain demographic groups such as those over 65 years old and/or those with underlying conditions such as diabetes, lung diseases, cancer, immunodeficiency, heart disease, hypertension, asthma, kidney disease, and gastrointestinal tract and liver disease [10,11,12]. Additionally, while it was reported during the early pandemic that young people were far less likely to be infected by SARS-CoV-2 and had very low mortality rates, there have been newer studies noting the rise of COVID-19 infections among the younger population in several jurisdictions since as early as April 2020 [13,14,15]. However, the potential temporal pattern(s) among the age groups have not been critically analyzed, especially at the global level.

Similarly, several reports have indicated that males are more likely to experience severe cases and higher mortality rates of COVID-19 than females [16,17,18,19]. For example, in a study conducted in China on the distribution of COVID-19 severe cases and case fatality rates, it was noted that males had significantly higher numbers of severe cases and case fatality rates than females [16]. A meta-analysis of global cases similarly revealed that males experienced more severe symptoms, with three times the odds of being admitted to intensive care units than females [17]. The underlying mechanism for potential sex-specific differences is unknown, but one suggested mechanism is that a potential link exists between high testosterone levels and high-level expression of angiotensin-converting enzyme 2 (ACE2), the receptor for the COVID-19 virus that is expressed on the membranes of cells in the lungs, arteries, heart, kidney, and intestines [20,21]. While males often experience more severe cases of COVID-19, it is unclear whether gender plays a role in susceptibility to COVID-19 infection. Published reports have shown conflicting results, with some stating that there is no difference in proportions between male and female COVID-19 infections, while others claim that females have a higher proportion of COVID-19 cases [16,17]. Additionally, at present, sex-based analyses have been largely focused on aggregate data at a specific time point. The potential global temporal patterns of COVID-19 infection and death between the two sexes remain largely unknown [22,23].

This paper aims to investigate the temporal patterns of age and sex distribution for COVID-19 cases, hospitalizations, and deaths across multiple countries where such data are available. Specifically, we obtained weekly COVID-19 case, hospitalization, and death data from 15 countries between 1 January 2020 and 1 January 2021, covering a total of 18,322,481 infected people and 612,172 deaths. The data were used to calculate: (i) the relative proportions of COVID-19 cases among individual age groups over time; (ii) the relative proportions of COVID-19 hospitalizations and deaths among age groups over time; (iii) the case fatality rates among age groups over time; (iv) the relative proportions of COVID-19 cases between the two sexes over time; (v) the relative proportions of COVID-19 hospitalizations and deaths between the two sexes over time; (vi) the case fatality rates between the two sexes over time; and (vii) the potential similarities and differences among countries in age group- and sex-based distributions. The data were analyzed to determine whether there were significant differences among age groups and between the two sexes in COVID-19 infections and deaths through the pandemic until the end of 2020.

## 2. Materials and Methods

We searched publicly available data related to COVID-19 for records between 1 January 2020 and 1 January 2021. From those data sources, each country’s data were checked for potential inclusion into our analyses based on the following four criteria.

1. The data source must contain national COVID-19 case data and/or hospitalization data and/or death data.
2. The data must be reported following the same criteria across regions within each country and must include either daily reports, weekly reports, or seven-day rolling averages, and contain age group information. Sex was extracted from the data if available.
3. The data must have at least four numerical data points or a month’s worth of data.
4. The country must have a population size of at least 1,000,000 to include sufficient data points of COVID-19 cases for inclusion in analysis.

Approximately 100 countries had publicly available epidemiological data on COVID-19 in 2020. However, most countries were excluded from our analyses due to not meeting one or more of the above four criteria. Data from 15 countries met all inclusion criteria. Those 15 countries were Brazil, Canada, Chile, Germany, India, Italy, Netherlands, New Zealand, Peru, Portugal, South Africa, South Korea, Turkey, United Kingdom, and the United States. These countries were from all six permanently inhabited continents. The data were extracted and filtered to include date of diagnosis, date of death, patient age, and sex if available. General population demographic data, specifically age and sex distribution for each country, was obtained through each country’s most recent census. The original data sources, data availability, and a summary of collected data for the 15 countries are presented in Supplementary Table S1. Aggregation of data by week, sex, and age brackets was performed using R. Data without defined age groups, but which included the specific ages of individuals were assigned into the following 10-year age groups: 0–9 years, 10–19 years, 20–29 years, 30–39 years, 40–49 years, 50–59 years, 60–69 years, 70–79 years, and 80+ years. For our analyses, data from each country were arranged into male and female cases, hospitalizations, and deaths distributed across the age groups.

### 2.1. Data Analysis

The curated dataset, as described above, was used to analyze trends about age group and sex distributions in COVID-19 cases, hospitalizations, and/or deaths. The raw dataset and computer code and software used are made publicly available in the data availability statement. To achieve the objectives of this paper, we conducted two types of analyses. The first was to determine whether there were statistically significant differences between the two sexes and among the age groups in the relative proportion of COVID-19 cases, hospitalizations, deaths, and case fatality rates. The second was to determine whether there was any age group- or sex-specific temporal trends within each country during the pandemic. Below we describe the specific analyses.

### 2.2. Differences between Age Groups and Sexes

Data for COVID-19 cases, hospitalizations, and deaths were first represented as proportions, with each proportion calculated as the number of cases for a specific age group divided by the total census population of that age group for that country. Furthermore, we standardized each week’s total proportions to 100% to make the data comparable between weeks. For our paper, this data is referred to as ‘relative proportion data’. In addition, data for COVID-19 deaths were further calculated to reflect case fatality rates. These were calculated as the total number of deaths for a given week divided by the total number of cases for the same week for each age group of each sex. Case hospitalization rates were not calculated due to the large number of missing data in most countries. All data used in each calculation were the total incident data for each 7-day period; and they were not cumulative.

A two-factorial ANOVA test (the two factors were age groups as reported by the country and sex) was performed on the relative proportion data for each country to examine if statistically significant differences were present among age groups and between the two sexes. ANOVA is a statistical test used commonly for hypothesis testing and requires multiple independent observations. In our analyses, the weekly observations (i.e., the relative proportions) for each age group of each sex were treated as independent data points. A two-factorial ANOVA tests for significant differences stratified by two variables, in this case sex and age [24]. Sex was considered binary in this report. Each week’s data, separated by age group and sex, were used as separate independent observations for that age group and sex in each country.

To determine whether the weekly repeats in the relative proportion data were independent and met ANOVA criteria, a correlation analysis was performed. The relative proportion data from each age group in each country over time were used to identify potential correlations, using the Pearson correlation test. If the weekly data are temporally correlated for each age group within each country, we would expect statistically significant correlations for all age groups. In contrast, the absence of correlation among data from different weeks over time would indicate the relative independence of weekly relative proportions of data. Our analyses revealed that overall, 126/226 (55.7%) of the correlations were non-significant, while 100/226 (44.3%) of the correlations were statistically significant. The significant correlations included both positive and negative correlations. A summary of the correlation analyses is presented in Supplementary Table S2.

Given a greater proportion of correlation as not significantly correlated, we proceeded to use the weekly numbers as independent measures of each age group and each sex to estimate the overall age group- and sex-based contributions to the total variations in COVID-19 prevalence in each country. Presence of statistically significant differences, as determined by the *p* value obtained from the ANOVA tests, would indicate that at least two distinct age groups or the two sexes had dissimilar means in the relative proportions of COVID-19 cases, hospitalizations, deaths, and/or case fatality. Statistically significant results between age groups as revealed by ANOVA were then further investigated using Tukey’s post-hoc test to explore which specific age groups had statistically significant differences.

To report the magnitude of variation among all age groups in each country, the F statistic value from ANOVA was used [25,26]. The F statistic is a mean square ratio of the between-group variance (i.e., variance among the age groups) and within group variance (i.e., variance within individual age groups). The F statistic is commonly used as a hypothesis test to identify differences in group means [27]. Statistically significant F values would indicate that not all group means are equal, but it does not identify which groups are significantly different from each other. To identify which age groups are statistically significantly different from each other, we employed Tukey’s post-hoc test that makes direct comparisons between groups [28]. In Tukey’s post-hoc test, the Q-stat measures the magnitude of difference between groups. The Q-stat value is also known as the studentized range distribution and represents the difference between the largest and smallest standard deviations from the group mean in the two groups compared [29]. Statistical significance of both the ANOVA and Tukey’s post-hoc tests follow those recommended in these tests [27,28,29].

ANOVA tests for all countries were performed in Excel and Google Sheets using the XLMiner Analysis ToolPak add-on [30]. Tukey’s post-hoc test was performed using the Real Statistics Resource Pack extension in Excel [31]. The F statistic values reported in Table 1 and Table 2, as well as the Q-stat values reported in Supplementary Tables S3–S7 were derived from these programs and analyses.

### 2.3. Patterns of Weekly Changes between Age Groups and Sexes

To investigate the potential temporal dynamics of COVID-19 among age groups and between the two sexes, we calculated the changes between consecutive weeks in the relative proportions of COVID-19 cases, hospitalizations, deaths, and case fatality rate. The objective was to investigate if certain age group(s) or either sex show consistent change, either increasing or decreasing relative to other group(s), for any of the COVID-19 indicators we investigated above, throughout the pandemic. The weekly changes were calculated as the difference between any week and the previous week over the entire pandemic. Thus, for each age group or each sex, there would be N-1 data points of difference between weeks, where N refers to the number of weeks that the original observed data were available. These weekly changes were then analyzed similar to those of the original relative proportion data using two-factorial ANOVA and Tukey’s post-hoc tests.

## 3. Results

Among the 15 countries, 13 (excluding Brazil and India) reported both age and sex information for our analyzed COVID-19 cases. Similarly, 13 of the 15 countries (excluding Chile and New Zealand) reported age and sex information for COVID-19 deaths. Among all the countries, three (Brazil, Chile, and India) reported age and sex information for COVID-19 hospitalizations. The detailed raw data is made publicly available in the data availability statement. Supplementary Table S1 details the most recent census data for each country used in our analysis. Below, we describe the relative proportions and distributions of COVID-19 among age groups and between the two sexes in COVID-19 cases, hospitalizations, and deaths within each of the 15 countries.

### 3.1. Variations in the Relative Proportions of COVID-19 Cases among Age Groups and between Sexes

Table 1 summarizes the ANOVA F values and the statistical significance of the F values regarding differences among age groups and between the two sexes in the relative COVID-19 case proportions in each of the 13 countries.

Based on the ANOVA test, when weekly data over the pandemic in 2020 were compared, all 13 of the countries displayed statistically significant differences in relative case proportions between age groups (Table 1). For all 13 countries showing significant differences, we further conducted a Tukey’s post-hoc test. The detailed values of Tukey’s post-hoc tests between age groups are shown in Supplementary Table S3. Here, based on the age group(s) with the highest relative proportions of COVID-19 cases, three patterns emerged. The first pattern included eight countries (Canada, Chile, Germany, New Zealand, Portugal, South Korea, Turkey, and the United States), which showed the highest relative COVID-19 frequencies in the 20–39 age group over the course of the pandemic. The second pattern was found in three countries (Italy, Netherlands, and the United Kingdom), which had the oldest age group (ages 80+) showing the highest relative proportions of cases over the pandemic. The third pattern was observed in two countries (Peru and South Africa), where the highest relative proportion of cases was found in the middle-aged group (ages 50–59).

Of the 13 countries, ten provided sex data for their COVID-19 cases. Between the two sexes, the ANOVA results showed that six of the countries displayed statistically significant differences between the two sexes. Specifically, Chile, Peru, and South Korea had significantly higher relative male case proportions than females. Conversely, Netherlands, United Kingdom, and the United States had significantly higher relative female case proportions than males. For the remaining four countries, there was no significant difference between the two sexes in the relative proportions of COVID-19 cases.

### 3.2. Variations in the Relative Proportions of COVID-19 Hospitalizations among Age Groups and between Sexes

Of the 15 countries, three (Brazil, Chile, and India) released data on hospitalizations with age group information included, while two (Chile and India) had sex information associated with the hospitalization data. The ANOVA results revealed that all three countries had statistically significant differences in relative hospitalization proportions among the age groups (Table 2). Tukey’s post-hoc test showed that in Brazil and Chile, the oldest age group (ages 80+) had the highest proportion of hospitalizations. However, in India, the younger individuals (ages 20–29) had the highest proportion of hospitalizations. The detailed values of Tukey’s post-hoc tests between age groups are shown in Supplementary Table S4. In both Chile and India, there was a significantly higher proportion of male hospitalizations than females throughout the pandemic.

### 3.3. Variations in the Relative Proportions of COVID-19 Deaths among Age Groups and between Sexes

Of the 15 countries, 13 provided weekly data on COVID-19 deaths with age group information included. The only countries missing that information were Chile and New Zealand. The ANOVA test results showed that all 13 countries had statistically significant differences among age groups in the relative proportions of deaths due to COVID-19 (Table 3). Thus, for all 13 countries, we conducted Tukey’s post-hoc test to identify the age group(s) with the highest COVID-19 death proportions. The detailed values of Tukey’s post-hoc tests between age groups are shown in Supplementary Table S5. Our results showed that in all 13 of the countries (Brazil, Canada, Germany, India, Italy, Netherlands, Peru, Portugal, South Africa, South Korea, Turkey, United Kingdom, and the United States), the highest proportion of deaths was found in their oldest age group. This ranged from 80+ years for some countries to 90+ for others, depending on the grouping of available age group data by different countries.

Of the nine countries with sex information associated with COVID-19 deaths, two (Canada and Netherlands) showed no statistically significant difference between the two sexes. In contrast, the remaining seven countries (India, Italy, Peru, Portugal, South Korea, United Kingdom, and the United States) had significantly higher proportions of deaths for males than females.

### 3.4. Variations in COVID-19 Case Fatality Rates among Age Groups and between Sexes

We calculated the case fatality rate for each age group in each country and investigated whether the age groups differed in case fatality rates during the pandemic within each country. Due to missing or mismatched age group and sex data in six of the 15 countries between the case and death data, only nine countries were included in case fatality analyses. The ANOVA results showed that, in all nine countries, there was an overall significant difference among age groups in case fatality rates (Table 4). The Tukey’s post-hoc test showed that in all countries, the older age groups experienced higher case fatality rates (Supplementary Table S6). However, there were differences among countries. Specifically, case fatality rates were significantly higher in individuals ages 50+ in Peru; ages 60+ in Germany, South Africa, and the United Kingdom; ages 65+ in Turkey; ages 70+ in Netherlands, Portugal, and South Korea; and ages 80+ in Italy compared to those at younger ages. However, beyond the stated ages (e.g., ages 70+ in Netherlands, Portugal, and South Korea) within each country, we did not see statistically significant differences between the age groups (i.e., no difference between the 70–79 age group and the 80+ age group in Netherlands, Portugal, and South Korea) (Supplementary Table S6).

The ANOVA results showed that four of the six countries with sex-stratified COVID-19 case fatality rates displayed statistically significant differences between the two sexes. Specifically, Netherlands, Peru, Portugal, and the United Kingdom had significantly higher male case fatality rates than females. Of the remaining two countries (Italy and South Korea), the case fatality rates were similar between males and females through the pandemic.

### 3.5. Weekly Changes in the Relative Proportions of COVID-19 Cases, Hospitalizations, and Deaths

Our analyses above showed significant differences among age groups and between the two sexes in COVID-19 cases, hospitalizations, and deaths in at least some countries. To further investigate whether the differences changed during the pandemic through 2020, we analyzed the weekly differences between successive weeks. If a majority of weeks showed increases or decreases in the relative proportions of COVID-19 cases, hospitalizations or deaths compared to previous weeks for a specific age group or sex during the pandemic, consistent overall positive or negative values, respectively, would be detected. However, our analyses showed that none of the weekly differences between the two sexes were statistically significant (Table 1, Table 2 and Table 3). Similarly, in all countries except the United Kingdom, there was no consistent increase or decrease in any age group in any of the COVID-19 indicators. Specifically, in the United Kingdom, the relative proportions of COVID-19 cases in those aged 80+ decreased through the pandemic. The detailed Tukey’s post-hoc values for the United Kingdom is shown in Supplementary Table S7.

## 4. Discussion

To the best of our knowledge, this longitudinal study represents one of the first attempts to analyze demographics for COVID-19 cases, hospitalizations, and deaths at a global level. Even though there has been much news surrounding this topic, the existing literature has focused on local or regional populations with relatively small sample sizes. In contrast, this study analyzed COVID-19 data from multiple countries where information on age and sex are available from the beginning to the end of 2020. Our findings provide novel information about the broad patterns of COVID-19 infections and deaths among age groups and between the two sexes. Below, we discuss the potential contributors to the observations and the implications of our results to managing the ongoing pandemic.

### 4.1. Effects of Age

Over the course of the pandemic, age groups have shown significant differences in their relative proportions of COVID-19 cases in all countries. Earlier studies of epidemiology patterns found the elderly to have the highest incidence of COVID-19, while those under 20 had low incidences of infection [9,10,11,12]. However, several later studies found individuals between 20–49 years to represent an increasing number of COVID-19 infections [14,15,32,33]. Such trends have been attributed to factors such as the need to work and working in essential jobs with more human contact [33]. However, it has also been suggested that this trend could be due to differences in COVID-19 testing access, rather than in the actual number of COVID-19 infections in the population [34,35]. Specifically, testing older adults was likely prioritized due to the more severe consequences they are faced with, likely contributing to their greater proportions in many countries, especially at the beginning of the pandemic when there were limited capacities in COVID-19 testing. With increasing testing capacities and the realization that a large number of asymptomatic cases in most communities may be spreading the virus, the younger age groups began to receive similar attention for testing, thus potentially contributing to the increasing number of cases for the younger and middle age groups. Our analyses are consistent with the hypothesis that all age groups are susceptible to COVID-19 infections. However, there are differences in the relative proportions among age groups throughout the pandemic, likely contributed by health and behavioral differences among age groups, and by testing capacity and policy differences within and among individual countries. The variations among countries in age groups with the highest case proportions suggest that individual countries should analyze their own demographic COVID-19 data in detail, with a focus on the relationships between infection rate and local policies, traditions, and/or customs and how those factors might affect the groups at highest risk of infection. For example, closing institutions such as public schools and universities could be beneficial in curbing COVID-19 cases for the younger generation. Monitoring long-term care facilities and enforcing social distancing policies could be helpful for protecting the elderly. 

Throughout the pandemic, older age groups have demonstrated more severe cases, leading to greater proportions of hospitalizations [8,9,10,11,12]. Our analysis agreed with this general pattern, with both Brazil and Chile showing their highest hospitalization proportions in their oldest age group. Of interest, however, was India, which showed their highest hospitalization proportion in those aged 20–29 yr. This could be due to strict stay at home orders for older adults in India or greater differences in socioeconomic status between the young and old in India [36].

Past studies have also reported that risk of death from COVID-19 increased with age, with COVID-19 case fatality rates increasing from 0.002% at age 10 to 15% at age 85 [37]. Our results are consistent with the pattern reported in earlier studies from individual jurisdictions in that patients at 80+ years experienced higher mortality rates than those of younger age groups. It is hypothesized that older adults typically experience worse outcomes due to physiological changes that come with aging, such as a decrease in the immune system’s functioning. Specifically, immunosenescence is a critical change associated with age, which could contribute to the increased fatality of elderly COVID-19 patients. Immunosenescence is caused by a decreased production of lymphocytes and the impaired function of innate immune cells, which adversely affects the ability of the immune system to recognize and clear viruses. Immunosenescence can also cause inflamm-aging, a persistent long-term activation of the innate immune system [38,39,40,41,42]. Tissue dysfunction and degeneration may result due to chronic low-grade inflammation. Additionally, older adults might experience greater fatality due to increased underlying comorbidities and health conditions associated with age. To reduce fatality due to COVID-19, continued protection of this vulnerable group should be a priority, including early vaccination, frequent testing, and screening of not only the vulnerable groups, but also those working with them [43].

### 4.2. Effects of Sex

In our analysis, no consistent pattern was found in the relative case proportions of COVID-19 between males and females, with some countries having a higher relative proportion of cases in males, and others having a higher relative proportion of cases in females. Our results are similar to previous local studies that reported that males and females overall have similar proportions of COVID-19 cases [12,44,45]. Disease prevalence is thought to be influenced by factors that affect risk of exposure and access to testing. A potential reason behind the lack of consistent patterns in disease prevalence between the sexes may be due to varying cultural roles and gender norms in each country [44]. With regards to risk of exposure to COVID-19, essential workers may be differently distributed across the sexes depending on the country of residence. Differences in gender equality between the countries might also affect the apparent proportion of infections between males and females, as gender inequality is hypothesized to be associated with the ability to receive a medical test. In a preliminary analysis by Tadiri et al., it was suggested that in countries with high gender inequality, men might be more likely to be exposed to COVID-19 as they are more likely to be employed [44]. As such, men may be more likely to receive COVID-19 tests, resulting in a higher proportion of male cases in countries of higher gender inequality. Our results similarly presented this finding with Chile and Peru, countries of higher gender inequality, exhibiting greater male case proportions; while the opposite was seen in Netherlands and the United Kingdom. However, it should be noted that there are likely interactions between several factors that differ between each country, contributing to differences in the sex with the highest proportion of COVID-19 cases between countries. Further research should be conducted in this field to investigate such potential interaction effects.

There were greater proportions of male COVID-19 deaths than females in most countries. Our results are consistent with the existing literature that males experienced higher COVID-19 case fatality rates [12,17,46]. Specifically, several past studies have reported that a greater proportion of males experienced hospitalizations and deaths than females. These differences could be due to a variety of social, cultural, behavioral, and biological factors between the two sexes [47,48,49,50]. Cross-national surveys have shown that females displayed higher threat perceptions in response to the pandemic and were more likely to adopt preventative measures [51]. Hand hygiene was more frequently practiced in females than males, and females were more likely to rapidly contact healthcare providers upon presentation of symptoms [52,53,54]. Biological factors in females, such as higher estrogen levels and stronger immune function during pregnancy and post-childbirth, have also been associated with decreased levels of COVID-19 fatality [52,55]. Other contributing factors such as differences in medical comorbidities, use of medications, occupation, and lifestyle (such as smoking and alcohol use) could have also played a role in the differences in fatalities [48]. For example, males have a higher prevalence of cardiovascular, hypertensive, and diabetic comorbidities than females, all of which are associated with COVID-19 deaths [54,56,57]. It has been observed that women smoke and drink less than men, this could be due to different social sanctions (i.e., low acceptance levels for females smoking and drinking) or parental monitoring [58,59]. Currently, more research needs to be conducted to evaluate sex- and gender-associated factors that underlie these comorbidities in the context of COVID-19 [57].

### 4.3. Weekly Changes in Cases, Hospitalizations, and Deaths

To our knowledge, this analysis is the first report of weekly differences representing a rate of change in COVID-19 cases, hospitalizations, and deaths through 2020. Results of our analysis suggested little to no evidence for consistent upward or downward changes for either sex or for any specific age group. A notable exception is the age difference in weekly changes in case proportions for the United Kingdom (*p* < 0.001). Specifically, in the United Kingdom, relative to those of younger populations, those in the 80+ age group had a decreasing proportion of COVID-19 cases over time through the pandemic. At present, the reasons for the consistent changes in this age group in the United Kingdom is not known. However, they are likely correlated to changes in public health policy.

### 4.4. Alternative Analyses

In this study, we focused on analyzing the relative proportions of COVID-19 cases, hospitalizations, and deaths among age groups and between the two sexes. In analyzing natural infectious disease outbreaks, due to the multiplicative nature of disease spread, it is common to analyze transformed case number or case proportion data to make the data suitable for ANOVA. In the case of COVID-19, because of government interventions and public health measures, aside from isolated communities, the disease did not spread at a rate close to its potential in any country. Instead, we saw stasis, and up and down fluctuations in the numbers of cases, hospitalizations, and deaths in most countries through 2020. By focusing on analyzing the relative proportions, we attempted to minimize the global weekly changes on our estimates of the relative differences among age groups and between the two sexes. However, to investigate whether analyzing transformed case data could influence our general conclusion, we log transformed the COVID-19 infection case data from three countries (Italy, UK, and USA) and performed ANOVA and Tukey’s post-hoc analyses. Overall, the ANOVA F values and the Tukey’s post-hoc Q -stat values were all smaller than our initial analyses, with few significant differences between age groups (Supplementary Table S8). This was expected because, compared to the initial analyses constraining each week’s proportions to 100% to minimize variation among weeks, the new analyses had large variations among weeks for each age group and each sex. However, in all three countries, age groups still showed significant differences in case numbers (Supplementary Table S8).

In the temporal analyses, we re-analyzed the log transformed case data in three countries (Italy, UK, and USA) using the formula [(N_t+1_) − N_t_] to investigate whether there was any consistent pattern of increase or decrease between weeks for any age group during 2020. Our results showed that only the very young age groups (males 0–9 yr and females 0–9 yr and 10–19 yr) in the US showed statistically significant decreases (Supplementary Table S9). In both Italy and UK (as well as other age groups in the US), we found no statistically significant consistent changes.

## 5. Conclusions, Limitations, and Perspectives

Our analyses identified that globally, age groups differed significantly in the relative proportions of COVID-19 cases, hospitalizations, and deaths. In addition, we observed differences among countries in each of the COVID-19 indicators. Similarly, depending on the country and COVID-19 indicator, the two sexes may also show significant differences. Overall, we saw limited evidence for consistent temporal change among age groups or between the two sexes during the pandemic.

Despite the large sample sizes over many countries, our study has several limitations. First, although an effort was made to include countries from various parts of the world in order to capture a wider perspective, data were only publicly available for a small sample of countries. As such, trends may not be generalizable to the world. Second, the timeline over which data were reported varied between countries and often did not cover the entire timeline of the pandemic. Third, available databases from many countries often did not include sex and age data for every case that occurred during the time period. Hence, many cases and deaths may have been excluded due to a lack of age and sex information. Fourth, different countries used different age groups in their reporting, making direct comparisons between many countries impossible.

In addition, there are likely some differences in testing approaches between countries due to population distributions, availability of laboratory tests, and reporting between medical systems. Countries where testing was done primarily for those who exhibit severe symptoms and seek care are likely to disproportionately identify cases in older individuals. Testing strategies may have also shifted during the pandemic, affecting the consistency of the reported data. Estimates conducted for case fatality rate rely solely on publicly available data that may not reflect actual values and may not consider undocumented deaths from COVID-19. Attribution of deaths to COVID-19 may have led to bias for certain age groups if countries defined COVID-19 deaths differently. Current policies and pandemic restrictions can also indirectly affect the fatality rate through delayed emergency care, on-going health issues, or affected social determinants to health (i.e., socioeconomic status) [39,60,61]. Differences in healthcare systems, as well as the effectiveness and accuracy of a country’s COVID-19 testing approaches may also affect the reported data and case fatality rates between age groups.

Overall, it should be cautioned that our understanding of age and sex influences on disease prevalence and outcomes for COVID-19 is greatly limited by numerical comparisons alone, in addition to the fact that such comparisons were already limited by the quality and availability of data. As well, ANOVA was the primary statistical method used in this analysis of relative proportion data. Our analyses may also be limited by the potential issue of non-independence of the weekly data as seen in some age groups of some countries. Alternative approaches should be explored that could help identify additional patterns. It should also be acknowledged that the countries which were compared have different sociological traits, cultures, behaviors, laws, and health traditions. All of which could have contributed to the observed variations. While our analyses identified a number of broad patterns, further research should be conducted within individual countries among jurisdictions to investigate the potential influences of socioeconomical factors on COVID-19 prevalence and outcomes for COVID-19 to better help mitigate this and potential future pandemics.

## Figures and Tables

**Table 1:** Summary F values for variations among age groups and between the two sexes in relative proportions of COVID-19 cases and in weekly changes between successive weeks in the relative proportions of COVID-19 cases in 13 countries.

Country	F Values of Variations in Age Group and Sex Distributions in Relative Proportions of COVID-19 Cases			F Values of Variations in Age Group and Sex Distributions in Weekly Changes in Relative Proportions of COVID-19 Cases		
	Age Group	Sex	Number of Weeks	Age Group	Sex	Number of Data Points
Canada	7.007 ****	0.019 ^NS^	52	0.135 ^NS^	0.188 ^NS^	51
Chile	788.054 ****	67.963 ****	30	0.164 ^NS^	0.215 ^NS^	29
Germany	7.632 ****	NT	50	0.106 ^NS^	NT	49
Italy	39.586 ****	1.637 ^NS^	41	0.228 ^NS^	0.413 ^NS^	40
Netherlands	14.901 ****	12.748 ***	53	0.216 ^NS^	0.210 ^NS^	52
New Zealand	29.534 ****	1.180 ^NS^	46	0.163 ^NS^	0.000 ^NS^	45
Peru	194.779 ****	57.971 ****	43	1.132 ^NS^	0.427 ^NS^	42
Portugal	63.905 ****	1.591 ^NS^	20	0.505 ^NS^	0.017 ^NS^	19
South Africa	276.079 ****	NT	35	0.079 ^NS^	NT	34
South Korea	16.890 ****	15.297 ***	17	0.101 ^NS^	0.282 ^NS^	16
Turkey	1096.683 ****	NT	17	0.464 ^NS^	NT	16
UK	36.704 ****	5.768 *	21	3.703 ***	0.083 ^NS^	20
USA	186.231 ****	6.913 **	42	0.683 ^NS^	0.538 ^NS^	41

Values in Table 1 are F values. *p* values are indicated by * for <0.05, ** for <0.01, *** for <0.001, and **** for <0.0001. ^NS^ for not significant, ^NT^ for not tested.

**Table 2:** Summary F values for variations among age groups and between the two sexes in relative proportions of COVID-19 hospitalizations and in weekly changes between successive weeks in the relative proportions of COVID-19 hospitalizations in three countries.

Country	F Values of Variations in Age Group and Sex Distributions in Relative Proportions of COVID-19 Hospitalizations			F Values of Variations in Age Group and Sex Distributions in Weekly Changes in Relative Proportions of COVID-19 Hospitalizations		
	Age Group	Sex	Number of Weeks	Age Group	Sex	Number of Data Points
Brazil	10,221.786 ****	NT	17	0.012 ^NS^	NT	16
Chile	1682.793 ****	705.500 ****	30	0.026 ^NS^	0.018 ^NS^	29
India	12.364 ****	23.272 ****	33	0.457 ^NS^	0.544 ^NS^	32

Values in Table 2 are F values. *p* values are indicated by **** for <0.0001. ^NS^ for not significant, ^NT^ for not tested.

**Table 3:** Summary F values for variations among age groups and between the two sexes in relative proportions of COVID-19 deaths and in weekly changes between successive weeks in the relative proportions of COVID-19 deaths in 13 countries.

Country	F Values of Variations in Age Group and Sex Distributions in Relative Proportions of COVID-19 Deaths			F Values of Variations in Age Group and Sex Distributions in Weekly Changes in Relative Proportions of COVID-19 Deaths		
	Age Group	Sex	Number of Weeks	Age Group	Sex	Number of Data Points
Brazil	8322.494 ****	NT	17	0.718 ^NS^	NT	16
Canada	94.433 ****	2.698 ^NS^	52	0.020 ^NS^	0.005 ^NS^	51
Germany	378.249 ****	NT	50	0.586 ^NS^	NT	49
India	102.107 ****	88.581 ****	48	0.077 ^NS^	0.029 ^NS^	47
Italy	621.266 ****	17.179 ****	41	0.088 ^NS^	0.086 ^NS^	40
Netherlands	141.059 ****	3.422 ^NS^	52	0.047 ^NS^	0.004 ^NS^	51
Peru	638.911 ****	366.991 ****	43	0.418 ^NS^	0.029 ^NS^	42
Portugal	707.354 ****	78.234 ****	20	0.007 ^NS^	0.352 ^NS^	19
South Africa	673.015 ****	NT	33	0.004 ^NS^	NT	32
South Korea	45.412 ****	4.518 *	17	0.002 ^NS^	0.000 ^NS^	16
Turkey	2150.954 ****	NT	17	0.181 ^NS^	NT	16
UK	3965.551 ****	133.350 ****	20	0.040 ^NS^	0.295 ^NS^	19
USA	7072.619 ****	521.710 ****	42	0.243 ^NS^	0.686 ^NS^	41

Values in Table 3 are F values. *p* values are indicated by * for <0.05, and **** for <0.0001. ^NS^ for not significant, ^NT^ for not tested.

**Table 4:** Summary F values for variations among age groups and between the two sexes in COVID-19 case fatality rates in nine countries.

Country	Age Group F Value	Sex F Value	Number of Weeks
Germany	82.205 ****	NT	50
Italy	14.176 ****	3.197 ^NS^	41
Netherlands	57.551 ****	12.677 ***	52
Peru	265.975 ****	67.448 ****	43
Portugal	66.886 ****	11.967 ***	20
South Africa	46.113 ****	NT	33
South Korea	12.374 ****	0.0002 ^NS^	17
Turkey	67.084 ****	NT	17
UK	139.055 ****	13.535 ***	20

Values in Table 4 are F values. *p* values are indicated by, *** for <0.001, and **** for <0.0001. ^NS^ for not significant, ^NT^ for not tested.

## Data Availability

Publicly available datasets were analyzed in this study. This data can be found here: https://github.com/frankcsquared/covid-19-age-sex-data (accessed on 1 June 2021).

## Supplementary Materials

The following are available online at https://www.mdpi.com/article/10.3390/idr13020054/s1, Table S1: Summary data sources, data availability, and collected data for 15 countries included in analyses, Table S2: Summary of case data independence for 15 countries included in analyses, Table S3: Tukey’s post-hoc tests of pairwise age group comparisons in COVID-19 case proportions within each of 13 countries, Table S4: Tukey’s post-hoc tests of pairwise age group comparisons in COVID-19 hospitalization proportions within each of three countries, Table S5: Tukey’s post-hoc tests of pairwise age group comparisons in COVID-19 death proportions within each of 13 countries, Table S6: Tukey’s post-hoc tests of pairwise age group comparisons in COVID-19 case fatality rates within each of nine countries, Table S7: Tukey’s post-hoc tests of pairwise age group comparisons in the weekly changes of case proportions caused by COVID-19 in one country that showed significant differences between at least two age groups, Table S8: ANOVA and Tukey’s results performed on logit transformed case data compared to relative proportion data, Table S9: Temporal patterns of COVID-19 infection cases using the formula of N_t+1_/N_t_ between weeks.
