# Supplementary material for: Global Temporal Patterns of Age Group and Sex Distributions of COVID-19
Source: Infect Dis Rep. 2021 Jun 21;13(2):582–96. doi: 10.3390/idr13020054 (PMC8293195; doi:10.3390/idr13020054)
Supplement: Supplementary file 1 [file idr-13-00054-s001.zip › idr-1233434-supplementary.pdf]

Table S1. Summary data sources, data availability, and collected data for 15 countries included in analyses.

Country	Population size	Data source (weblink)	Time period of data availability	Total COVID-19 infections from Worldometers (our collected data)	Total COVID-19 deaths from Worldometers (our collected data)
Brazil	210,147,125	https://www.saude.gov.br/boletins-epidemiologicos	Jun 14, 2020 - Oct 10, 2020	5,091,840 (hospitalizations: 326,097)	150,236 (107,726)
Canada	37,971020	https://resources-covid19canada.hub.arcgis.com/datasets/compiled-covid-19-case-details-canada/data	Jan 1, 2020 - Dec 29, 2020	565,506 (274,038)	15,378 (4,477)
Chile	17,574,003	https://www.minsal.cl/nuevo-coronavirus-2019-ncov/informe-epidemiologico-covid-19/	May 24, 2020 - Dec 23, 2020	590,914 (591,168)	16,288 (no death data)
Germany	83,149,300	https://www.kaggle.com/headsortails/covid19-tracking-germany	Jan 28, 2020 - Jan 1, 2021	1773,540 (522,550)	34,859 (23,020)
India	1,326,093,247	https://github.com/covid19india/api	Jan 26, 2020 - Dec 21, 2020	10,075,422 (hospitalizations: 105,251)	146,145 (10,210)
Italy	60,317,116	https://www.epicentro.iss.it/coronavirus/aggiornamenti	Mar 13, 2020 - Dec 29, 2020	2,067,487 (2,036,877)	73,029 (70,428)
Netherlands	17,418,808	https://data.rivm.nl/covid-19/	Jan 1, 2020 - Dec 31, 2020	796,981 (787,617)	11,529 (11,210)
New Zealand	4,942,500	https://www.health.govt.nz/our-work/diseases-and-conditions/covid-19-novel-coronavirus/covid-19-current-situation/covid-19-current-cases/covid-19-current-cases-details	Feb 26, 2020 - Aug 30, 2020	1729 (1,306)	22 (no death data)

Peru	32,824,358	https://www.minsa.gob.pe/datosabiertos/	Mar 18, 2020 - Dec 29, 2020	1,010,496 (949,373)	37,574 (32,499)
Portugal	10,295,909	https://covid19.min-saude.pt/relatorio-de-situacao/	Mar 25, 2020 - Aug 11, 2020	56,499 (50,504)	1,761 (1,728)
South Africa	69,622,350	https://www.nicd.ac.za/wp-content/uploads/2020/05/2020-04-30-COVID-19WklyEpiBriefFinal_Week18.pdf	Apr 20, 2020 - Dec 26, 2020	994,911 (845,989)	26,973 (14,605)
South Korea	51,270,000	https://www.cdc.go.kr/board/board.es?mid=a30501000000&bid=0031&cg_code=C04	Mar 29, 2020 - Aug 8, 2020	14,562 (4,357)	304 (151)
Turkey	83,154,997	https://sbsgm.saglik.gov.tr/TR,66560/haftalik-rapor--weekly-report.html	Jun 29, 2020 - Oct 25, 2020	984,114 (164,516)	9,799 (4702)
United Kingdom	55,268,100	https://www.gov.uk/government/publications/national-covid-19-surveillance-reports	May 7, 2020 - Sep 29, 2020	454,968 (221,312)	42,125 (16,546)
United States	328,239,523	https://www.cdc.gov/coronavirus/2019-ncov/covid-data/covidview/07242020/clinical-labs.html; https://www.cdc.gov/coronavirus/2019-ncov/covid-data/covidview/07242020/public-health-lab.html; https://www.cdc.gov/coronavirus/2019-ncov/covid-data/covidview/07242020/commercial-labs.html; https://gis.cdc.gov/grasp/COVIDNet/COVID19_5.html	Mar 1, 2020 - Dec 26, 2020	19,518,373 (11,441,526)	345, 351 (314,870)

	Population Data Used	Year Collected of Population Data Used	Date of Most Recent Publicly Available National Census
Canada	https://www150.statcan.gc.ca/t1/tbl1/en/tv.action?pid=1710000501&pickMembers%5B0%5D=1.1&pickMembers%5B1%5D=2.2	2020	2016
Chile	https://population.un.org/wpp/Download/Standard/Population/	2019	2017
Germany	https://population.un.org/wpp/Download/Standard/Population/	2019	2011
Italy	https://population.un.org/wpp/Download/Standard/Population/	2019	2011
Netherlands	https://population.un.org/wpp/Download/Standard/Population/	2019	2011
New Zealand	https://population.un.org/wpp/Download/Standard/Population/	2019	2018
Peru	https://population.un.org/wpp/Download/Standard/Population/	2019	2017
Portugal	https://population.un.org/wpp/Download/Standard/Population/	2019	2011
South Africa	https://population.un.org/wpp/Download/Standard/Population/	2019	2011
South Korea	https://population.un.org/wpp/Download/Standard/Population/	2019	2019
Turkey	https://population.un.org/wpp/Download/Standard/Population/	2019	2020
USA	https://population.un.org/wpp/Download/Standard/Population/	2019	2020
UK	https://population.un.org/wpp/Download/Standard/Population/	2019	1921

Table S2. Summary of case data independence for 15 countries included in analyses
P values are indicated by * for <0.05/n, where n is the number of age groups for that specific country

Brazil

	0-5 yr	6-19 yr	20-29 yr	30-39 yr	40-49 yr	50-59 yr	60-69 yr	70-79 yr	80-89 yr	90+ yr
**Hospitalizations**	**0.56**	**0.51**	**-0.26**	**-0.24**	**-0.06**	**0.05**	**0.33**	**0.13**	**0.25**	**-0.27**

Canada

	0-19 yr	20-29 yr	30-39 yr	40-49 yr	50-59 yr	60-69 yr	70-79 yr	80+ yr
Male	0.93*	0.61*	0.66*	0.44*	-0.35	-0.16	-0.16	-0.39*
Female	0.87*	0.17	0.47*	0.46*	0.00	-0.27**	-0.30*	-0.25

Chile

	0-9 yr	10-19 yr	20-29 yr	30-39 yr	40-49 yr	50-59 yr	60-69 yr	70-79 yr	80+ yr
Male	0.69*	0.86*	-0.05	-0.57*	-0.70*	-0.64*	-0.62*	-0.33	-0.42
Female	0.68*	0.86*	0.35	-0.21	-0.13	-0.25	0.42	0.32	-0.34

Germany

	0-4 yr	5-14 yr	15-34 yr	35-59 yr	60-79 yr	80+ yr
Cases	0.09	0.89*	-0.10	-0.64*	0.12	-0.22

India

	0-9 yr	10-19 yr	20-29 yr	30-39 yr	40-49 yr	50-59 yr	60-69 yr	70-79 yr	80+ yr
Male	0.45	0.42	0.43	0.45	0.43	0.59*	0.29	0.19	0.01
Female	0.18	0.36	-0.19	0.82*	0.75*	0.81*	0.38	-0.06	0.36

Italy

	0-9 yr	10-19 yr	20-29 yr	30-39 yr	40-49 yr	50-59 yr	60-69 yr	70-79 yr	80-89 yr	90+ yr
Male	0.69*	0.66*	0.38	0.46*	0.58*	0.58*	0.05	-0.33	-0.61*	-0.54*
Female	0.71*	0.72*	0.55*	0.67*	0.76*	0.49*	0.42	-0.29	-0.69*	-0.62*

Netherlands

	0-9 yr	10-19 yr	20-29 yr	30-39 yr	40-49 yr	50-59 yr	60-69 yr	70-79 yr	80-89 yr	90+ yr
Male	0.36	0.87*	0.59*	0.67*	0.47*	0.50*	-0.11	-0.23	-0.19	-0.06
Female	0.44*	0.86*	0.49*	0.67*	-0.15	-0.15	0.10	-0.20	-0.13	

										-0.28

New Zealand

	0-9 yr	10-19 yr	20-29 yr	30-39 yr	40-49 yr	50-59 yr	60-69 yr	70-79 yr	80-89 yr	90+ yr
Male	-0.03	0.34	0.33	0.29	-0.14	-0.03	-0.18	-0.27	-0.34	-0.20
Female	0.30	-0.01	0.20	-0.08	-0.22	-0.11	-0.29	-0.15	-0.25	-0.25

Peru

	0-9 yr	10-19 yr	20-29 yr	30-39 yr	40-49 yr	50-59 yr	60-69 yr	70-79 yr	80+ yr
Male	0.21	0.81*	-0.42*	0.15	-0.77*	-0.75*	-0.32	-0.31	0.13
Female	0.77*	0.59*	0.57*	0.62*	0.41	0.26	-0.10	-0.09	0.62*

Portugal

	0-9 yr	10-19 yr	20-29 yr	30-39 yr	40-49 yr	50-59 yr	60-69 yr	70-79 yr	80+ yr
Male	0.85*	0.92*	0.59	0.48	0.36	-0.05	-0.33	-0.41	-0.75*
Female	0.91*	0.91*	0.59	0.37	-0.46	-0.86*	-0.55	-0.51	-0.72*

South Africa

	0-9 yr	10-19 yr	20-29 yr	30-39 yr	40-49 yr	50-59 yr	60-69 yr	70-79 yr	80+ yr
Cases	-0.26	0.53*	-0.24	-0.77*	-0.68*	0.23	0.83*	0.75*	-0.16

South Korea

	0-9 yr	10-19 yr	20-29 yr	30-39 yr	40-49 yr	50-59 yr	60-69 yr	70-79 yr	80+ yr
Male	0.47	-0.32	-0.28	0.49	0.61	0.67*	0.51	0.11	-0.26
Female	0.19	-0.42	-0.78*	-0.56	-0.38	0.33	0.40	0.46	-0.02

Turkey

	0-14 yr	15-24 yr	25-49 yr	50-64 yr	65-79 yr	80+ yr
Cases	-0.725*	0.40	0.30	0.60	-0.15	-0.19

United Kingdom

	0-4 yr	5-9 yr	10-19 yr	20-29 yr	30-39 yr	40-49 yr	50-59 yr	60-69 yr	70-79 yr	80+ yr
Male	0.79*	0.84*	0.97*	0.93*	0.79*	0.69*	0.53	-0.43	-0.81*	-0.88*
Female	0.75*	0.73*	0.97*	0.89*	0.71*	0.30	-0.08	-0.51	-0.87*	-0.90*

United States

	0-9 yr	10-19 yr	20-29 yr	30-39 yr	40-49 yr	50-59 yr	60-69 yr	70-79 yr	80-89 yr
Male	0.86*	0.83*	0.53*	-0.26	-0.62*	-0.67*	-0.60*	-0.56*	-0.67*
Female	0.86*	0.80*	0.54*	0.38	0.12	-0.51*	-0.39	-0.39	-0.53*

Table S3. Tukey's post-hoc tests of pairwise age-group comparisons in COVID-19 case proportions within each of 13 countries.
Values in Table are Q-stat. P values are indicated by * for <0.05, ** for <0.01, *** for <0.001, and **** for <0.0001.

Italy

	0-9 yr	10-19 yr	20-29 yr	30-39 yr	40-49 yr	50-59 yr	60-69 yr	70-79 yr	80-89 yr
10-19 yr	4.31****								
20-29 yr	11.70****	7.39****							
30-39 yr	8.49****	4.19	3.21						
40-49 yr	6.95****	2.64	4.76*	1.55					
50-59 yr	7.15*	2.84	4.55*	1.35	0.22				
60-69 yr	4.69*	0.38	7.01****	3.81	2.26	2.46			
70-79 yr	4.56****	0.25	7.15****	3.94	2.39	2.59	0.13		
80-89 yr	10.58****	6.27***	1.121	2.09	3.64	3.44	5.90**	6.03***	
90+ yr	23.29****	18.98****	11.58****	14.79****	16.34****	16.14****	19.60****	18.73****	12.70****

UK

	0-4 yr	5-9 yr	10-19 yr	20-29 yr	30-39 yr	40-49 yr	50-59 yr	60-69 yr	70-79 yr
5-9 yr	0.01								
10-19 yr	4.70*	4.69*							
20-29 yr	14.21****	14.20****	9.51****						
30-39 yr	10.45****	10.45****	5.75**	3.755					
40-49 yr	8.92****	8.91****	4.22	5.287**	1.53				
50-59 yr	7.10****	7.09****	2.40	7.113****	3.36	1.83			
60-69 yr	4.11	4.11	0.59	10.095****	6.34***	4.81*	2.98		
70-79 yr	3.25	3.24	1.45	10.961****	7.21****	5.67**	3.85	0.87	
80+ yr	18.72****	18.71****	14.02****	4.512****	8.27****	9.8****	11.63****	14.61****	15.47****

South Korea

	0-9 yr	10-19 yr	20-29 yr	30-39 yr	40-49 yr	50-59 yr	60-69 yr	70-79 yr

10-19 yr	4.33							
20-29 yr	15.06****	10.73****						
30-39 yr	9.13****	4.8*	5.92**					
40-49 yr	5.2**	0.87	9.86****	3.94				
50-59 yr	5.16**	0.83	9.89****	3.97	0.03			
60-69 yr	6.54***	2.21	8.51****	2.59	1.35	1.38		
70-79 yr	4.87*	0.54	10.18****	4.26	0.32	0.29	1.67	
80+ yr	4.7*	0.37	10.36****	4.44*	0.5	0.46	1.85	0.17

Canada

	0-19 yr	20-29 yr	30-39 yr	40-49 yr	50-59 yr	60-69 yr	70-79 yr
20-29 yr	5.97***						
30-39 yr	3.02	2.95					
40-49 yr	2.05	3.93	0.97				
50-59 yr	4.85*	1.13	1.83	2.8			
60-69 yr	1.42	4.55*	1.6	0.62	3.42		
70-79 yr	2.17	8.14****	5.18**	4.21	7.01****	3.59	
80+ yr	0.43	5.54**	2.59	1.62	4.42*	0.99	2.59

USA

	0-9 yr	10-19 yr	20-29 yr	30-39 yr	40-49 yr	50-59 yr	60-69 yr	70-79 yr
10-19 yr	17.04****							
20-29 yr	41.71****	24.67****						
30-39 yr	37.21****	20.17****	4.5*					
40-49 yr	39.03****	22****	2.67	1.83				
50-59 yr	36.29****	19.25****	5.42**	0.92	2.75			
60-69 yr	26.25****	9.21****	15.46****	10.95****	12.78****	10.03****		
70-79 yr	22.66****	5.62**	19.05****	14.55****	16.38****	13.63****	3.6	

80+ yr	37.74****	20.7****	3.97	0.53	1.29	1.45	11.49****	15.08****

Germany

	0-4 yr	5-14 yr	15-34 yr	35-59 yr	60-79 yr
5-14 yr	2.39				
15-34 yr	3.14	5.53**			
35-59 yr	1.13	1.26	4.26*		
60-79 yr	4.9**	2.51	8.04****	3.77	
80+ yr	0.81	3.2	2.33	1.94	5.71***

South Africa

	0-9 yr	10-19 yr	20-29 yr	30-39 yr	40-49 yr	50-59 yr	60-69 yr	70-79 yr
10-19 yr	6.46***							
20-29 yr	20.48****	14.01****						
30-39 yr	32.63****	26.17****	12.15****					
40-49 yr	42.43****	35.97****	21.95****	9.80****				
50-59 yr	46.24****	39.78****	25.77****	13.61****	3.81			
60-69 yr	34.93****	28.47****	14.46****	2.3	7.5****	11.31****		
70-79 yr	31.9****2	25.46****	11.45****	0.71	10.51****	14.32****	3.01	
80+ yr	44.89****	38.43****	24.42****	12.26****	2.46	1.35	9.96****	12.97****

Portugal

	0-9 yr	10-19 yr	20-29 yr	30-39 yr	40-49 yr	50-59 yr	60-69 yr	70-79 yr
10-19 yr	0.01							
20-29 yr	20.44****	20.43****						
30-39 yr	17.30****	17.29****	3.15					
40-49 yr	9.95****	9.94****	10.49****	7.35****				
50-59 yr	8.55****	8.54****	11.89****	8.75****	1.40			
60-69 yr	3.55	3.54	16.89****	13.74****	6.40***	5.00*		
70-79 yr	1.66	1.65	18.79****	15.64****	8.29****	6.89****	1.90	

80+ yr	17.10****	17.09****	3.34	0.19	7.15****	8.55****	13.55****	15.44****

Chile

	0-9 yr	10-19 yr	20-29 yr	30-39 yr	40-49 yr	50-59 yr	60-69 yr	70-79 yr
10-19 yr	17.26****							
20-29 yr	79.38****	62.12****						
30-39 yr	82.49****	65.24****	3.12					
40-49 yr	69.16****	51.90****	10.22****	13.33****				
50-59 yr	60.56****	43.31****	18.81****	21.93****	8.60****			
60-69 yr	49.32****	32.06****	30.05****	33.17****	19.84****	11.24****		
70-79 yr	43.34****	26.09****	36.03****	39.15****	25.82****	17.22****	5.98***	
80+ yr	68.27****	51.01****	11.11****	14.23****	0.89	7.70****	18.94****	24.92****

Turkey

	0-14 yr	15-24 yr	25-49 yr	50-64 yr	65-79 yr
15-24 yr	52.95****				
25-49 yr	87.36****	34.41****			
50-64 yr	82.31****	29.36****	5.05**		
65-79 yr	79.80****	26.85****	7.56****	2.51	
80+ yr	75.84****	22.89****	11.52****	6.47****	3.96

Peru

	0-9 yr	10-19 yr	20-29 yr	30-39 yr	40-49 yr	50-59 yr	60-69 yr	70-79 yr
10-19 yr	3.98							
20-29 yr	26.30****	22.32****						
30-39 yr	28.57****	24.59****	2.28					
40-49 yr	33.05****	29.07****	6.76****	4.48*				
50-59 yr	36.35****	32.37****	10.05****	7.77****	3.29			
60-69 yr	34.51	30.53	8.22****	5.94***	1.46	1.83		

	****	****						
70-79 yr	34.95 ****	30.97 ****	8.65****	6.37***	1.89	1.400	0.43	
80+ yr	35.03 ****	31.05 ****	8.73****	6.46***	1.97	1.32	0.51	0.08

New Zealand

	0-9 yr	10-19 yr	20-29 yr	30-39 yr	40-49 yr	50-59 yr	60-69 yr	70-79 yr	80-89 yr
10-19 yr	1.17								
20-29 yr	11.56 ****	10.39* ***							
30-39 yr	10.28 ***	9.11****	1.28						
40-49 yr	1.15	0.02	10.41 ****	9.13****					
50-59 yr	0.34	0.83	11.21 ***	9.94****	0.81				
60-69 yr	1.08	2.25	12.63 ****	11.36****	2.23	1.42			
70-79 yr	3.01	4.18	14.57 ****	13.29****	4.16	3.35	1.93		
80-89 yr	3.80	4.97*	15.35 ****	14.08 ****	4.95*	4.14	2.72	0.79	
90+ yr	3.91	5.08*	15.47 ****	14.19 ****	5.06*	4.25	2/83	0.90	0.11

Netherlands

	0-9 yr	10-19 yr	20-29 yr	30-39 yr	40-49 yr	50-59 yr	60-69 yr	70-79 yr	80-89 yr
10-19 yr	5.61**								
20-29 yr	11.27 ****	5.66**							
30-39 yr	7.77 ****	2.16	3.50						
40-49 yr	9.86 ****	4.26	1.40	2.10					
50-59 yr	9.50 ****	3.89	1.77	1.74	0.36				

60-69 yr	6.01***	0.40	5.26**	1.76	3.86	3.50			
70-79 yr	4.99*	0.61	6.28*<br>**	2.77	4.87*	4.51*	1.01		
80-89 yr	7.19****	1.59	4.07	0.57	2.67	2.31	1.19	2.20	
90+ yr	13.98<br>****	8.37****	2.71	6.21***	4.11	4.47	7.97****	8.98****	6.78****

Table S4. Tukey's post-hoc tests of pairwise age-group comparisons in COVID-19 hospitalization proportions within each of three countries. Values in Table are Q-stat. P values are indicated by * for <0.05, ** for <0.01, *** for <0.001, and **** for <0.0001.

India

	0-9 yr	10-19 yr	20-29 yr	30-39 yr	40-49 yr	50-59 yr	60-69 yr	70-79 yr
10-19 yr	1.21							
20-29 yr	9.65****	8.44****						
30-39 yr	8.33****	7.12****	1.32					
40-49 yr	8.13****	6.92****	1.52	0.20				
50-59 yr	9.32****	8.11****	0.33	0.99	1.19			
60-69 yr	8.20****	6.99****	1.45	0.13	0.07	1.12		
70-79 yr	5.87**	4.65*	3.79	2.47	2.27	3.46	2.34	
80+ yr	4.96*	3.74	4.70*	3.38	3.17	4.36	3.25	0.91

Chile

	0-4 yr	5-17 yr	18-49 yr	50-59 yr	60-69 yr	70-79 yr
5-17 yr	7.05****					
18-49 yr	1.93	8.98****				
50-59 yr	22.70****	15.64****	24.62****			
60-69 yr	41.13****	34.07****	43.05****	18.43****		
70-79 yr	65.35****	58.30****	67.28****	42.66****	24.22****	
80+ yr	109.95****	102.89****	111.87****	87.25****	68.82****	44.59****

Brazil

	0-5 yr	6-19 yr	20-29 yr	30-39 yr	40-49 yr	50-59 yr	60-69 yr	70-79 yr	80-89 yr
6-19 yr	2.97								
20-29 yr	1.05	4.02							
30-39 yr	9.08****	12.05****	8.03****						
40-49 yr	18.60****	21.57****	17.55****	9.52****					
50-59 yr	33.11****	36.08****	32.06****	24.03****	14.51****				
60-69 yr	59.61****	62.58 ****	58.57 ****	50.53 ****	41.01 ****	26.50****			
70-79 yr	106.90****	109.87 ****	105.85 ****	97.81 ****	88.29 ****	73.79 ****	47.28 ****		

80-89 yr	190.87****	193.84 ****	189.82 ****	181.79 ****	172.27 ****	157.76 ****	131.26 ****	83.97 ****	
90+ yr	300.77****	303.74 ****	299.72 ****	291.69 ****	282.17 ****	267.66 ****	241.15 ****	193.87 ****	109.90****

Table S5. Tukey's post-hoc tests of pairwise age-group comparisons in COVID-19 death proportions within each of 13 countries. Values in Table are Q-stat. P values are indicated by * for <0.05, ** for <0.01, *** for <0.001, and **** for <0.0001.

Italy

	0-9 yr	10-19 yr	20-29 yr	30-39 yr	40-49 yr	50-59 yr	60-69 yr	70-79 yr	80-89 yr
10-19 yr	0.00								
20-29 yr	0.02	0.02							
30-39 yr	0.08	0.08	0.06						
40-49 yr	0.34	0.34	0.32	0.26					
50-59 yr	0.99	0.99	0.97	0.90	0.65				
60-69 yr	3.24	3.25	3.22	3.16	2.91	2.26			
70-79 yr	10.55****	10.56****	10.54****	10.47****	10.22****	9.57****	7.31****		
80-89 yr	31.42 ****	31.42 ****	31.40 ****	31.33 ****	31.08 ****	30.43 ****	28.17 ****	20.86 ****	
90+ yr	77.62 ****	77.63 ****	77.60 ****	77.54 ****	77.29 ****	76.64 ****	74.38 ****	67.07 ****	46.21****

UK

	0-4 yr	5-9 yr	10-19 yr	20-29 yr	30-39 yr	40-49 yr	50-59 yr	60-69 yr	70-79 yr
5-9 yr	0.13								
10-19 yr	0.11	0.02							
20-29 yr	0.01	0.14	0.11						
30-39 yr	0.29	0.42	0.40	0.29					
40-49 yr	1.74	1.87	1.85	1.73	1.45				
50-59 yr	4.81*	4.94*	4.92*	4.81*	4.52*	3.07			
60-69 yr	14.49****	14.62****	14.60****	14.48****	14.20****	12.75****	9.68****		
70-79 yr	40.94****	41.07****	41.05****	40.93****	40.65****	39.20****	36.13****	26.45****	
80+ yr	201.85 ****	201.97 ****	201.95 ****	201.84 ****	201.55 ****	200.11 ****	197.03 ****	187.35 ****	160.90****

South Korea

	0-9 yr	10-19 yr	20-29 yr	30-39 yr	40-49 yr	50-59 yr	60-69 yr	70-79 yr
10-19 yr	0.00							
20-29 yr	0.00	0.00						

30-39 yr	0.03	0.03	0.03					
40-49 yr	0.11	0.11	0.11	0.08				
50-59 yr	0.17	0.17	0.17	0.14	0.06			
60-69 yr	1.74	1.74	1.74	1.70	1.62	1.56		
70-79 yr	6.71***	6.71***	6.71***	6.67***	6.59***	6.53***	4.97*	
80+ yr	20.21****	20.21****	20.21****	20.18****	20.10****	20.04****	18.48****	13.51****

Canada

	0-19 yr	20-29 yr	30-39 yr	40-49 yr	50-59 yr	60-69 yr	70-79 yr
20-29 yr	0.04						
30-39 yr	0.13	0.10					
40-49 yr	0.56	0.52	0.42				
50-59 yr	2.06	2.02	1.93	1.50			
60-69 yr	4.28	4.24	4.15	3.72	2.22		
70-79 yr	12.42****	12.39****	12.29****	11.87****	10.37****	8.14****	
80+ yr	27.58****	27.55****	27.45****	27.02****	25.52****	23.30****	15.16****

USA

	0-4 yr	5-14 yr	15-24 yr	25-34 yr	35-44 yr	45-54 yr	55-64 yr	65-74 yr	75-84 yr
5-14 yr	0.05								
15-24 yr	0.15	0.20							
25-34 yr	0.87	0.91	0.71						
35-44 yr	2.48	2.52	2.32	1.61					
45-54 yr	6.96****	7.01****	6.81****	6.10***	4.49*				
55-64 yr	16.18****	16.23****	16.03****	15.32****	13.71****	9.22****			
65-74 yr	38.06****	38.11****	37.91****	37.20****	35.59****	31.10****	21.88****		
75-84 yr	94.65 ****	94.70 ****	94.50 ****	93.79 ****	92.18 ****	87.69 ****	78.47 ****	56.59****	
85+ yr	266.58 ****	266.63 ****	266.43 ****	265.72 ****	264.11 ****	259.62 ****	250.40 ****	228.52 ****	171.93****

India

	0-9 yr	10-19 yr	20-29 yr	30-39 yr	40-49 yr	50-59 yr	60-69 yr	70-79 yr
10-19 yr	0.03							
20-29 yr	0.23	0.26						
30-39 yr	0.84	0.88	0.62					
40-49 yr	2.91	2.95	2.69	2.07				
50-59 yr	7.67****	7.70****	7.44****	6.82****	4.75*			
60-69 yr	15.84****	15.87****	15.61****	14.99****	12.92****	8.17****		
70-79 yr	23.12****	23.16****	22.90****	22.28****	20.21****	15.46****	7.29****	
80+ yr	24.17****	24.20****	23.94****	23.32****	21.25****	16.50****	8.33****	1.04

Germany

	0-4 yr	5-14 yr	15-34 yr	35-59 yr	60-79 yr
5-14 yr	0.02				
15-34 yr	0.00	0.02			
35-59 yr	0.75	0.77	0.75		
60-79 yr	6.86****	6.88****	6.86****	6.11***	
80+ yr	48.70****	48.72****	48.70****	47.95****	41.84****

South Africa

	0-9 yr	10-19 yr	20-29 yr	30-39 yr	40-49 yr	50-59 yr	60-69 yr	70-79 yr
10-19 yr	0.03							
20-29 yr	0.50	0.47						
30-39 yr	1.94	1.92	1.44					
40-49 yr	6.56***	6.53***	6.05***	4.61*				
50-59 yr	18.11****	18.08****	17.61****	16.17****	11.56****			
60-69 yr	32.80****	32.77****	32.30****	30.85****	26.24****	14.69****		
70-79 yr	47.65****	47.62****	47.15****	45.70****	41.09****	29.53****	14.85****	
80+ yr	72.60****	72.57****	72.10****	70.65****	66.04****	54.49****	39.80****	24.95****

Portugal

	0-9 yr	10-19 yr	20-29 yr	30-39 yr	40-49 yr	50-59 yr	60-69 yr	70-79 yr
10-19 yr	0.00							

20-29 yr	0.07	0.07						
30-39 yr	0.41	0.41	0.34					
40-49 yr	0.51	0.51	0.44	0.10				
50-59 yr	1.73	1.73	1.66	1.32	1.22			
60-69 yr	6.28***	6.28***	6.21***	5.87**	5.77**	4.55*		
70-79 yr	17.62****	17.62****	17.55****	17.21****	17.10****	15.88****	11.34****	
80+ yr	81.23****	81.23****	81.16****	80.82****	80.72****	79.49****	74.95****	63.61****

Peru

	0-9 yr	10-19 yr	20-29 yr	30-39 yr	40-49 yr	50-59 yr	60-69 yr	70-79 yr
10-19 yr	0.07							
20-29 yr	0.38	0.31						
30-39 yr	1.16	1.09	0.78					
40-49 yr	3.82	3.75	3.44	2.66				
50-59 yr	10.47****	10.40****	10.09****	9.31****	6.65***			
60-69 yr	24.63****	24.55****	24.25****	23.46****	20.80****	14.16****		
70-79 yr	41.56****	41.49****	41.19****	40.40****	37.74****	31.09****	16.94****	
80+ yr	72.96****	72.89****	72.58****	71.80****	69.14****	62.49****	48.33****	31.39****

Brazil

	0-5 yr	6-19 yr	20-29 yr	30-39 yr	40-49 yr	50-59 yr	60-69 yr	70-79 yr	80-89 yr
6-19 yr	0.23								
20-29 yr	0.31	0.55							
30-39 yr	1.60	1.83	1.28						
40-49 yr	4.72*	4.95*	4.41	3.13					
50-59 yr	11.77****	12.01****	11.46****	10.18****	7.05****				
60-69 yr	31.37 ****	31.60 ****	31.05 ****	29.77 ****	26.64 ****	19.59 ****			
70-79 yr	71.77 ****	72.00 ****	71.45 ****	70.17 ****	67.04 ****	59.99 ****	40.40 ****		
80-89 yr	155.12 ****	155.35 ****	154.81 ****	153.52 ****	150.40 ****	143.35 ****	123.75 ****	83.35 ****	
90+ yr	273.46	273.69	273.15	271.86	268.74	261.68	242.09	201.69	118.34****

	****	****	****	****	****	****	****	****	

Turkey

	0-14 yr	15-24 yr	25-49 yr	50-64 yr	65-79 yr
15-24 yr	0.06				
25-49 yr	0.92	0.85			
50-64 yr	10.39****	10.33****	9.48****		
65-79 yr	49.14****	49.08****	48.23****	38.75****	
80+ yr	115.81****	115.75****	114.90****	105.42****	66.67****

Netherlands

	0-9 yr	10-19 yr	20-29 yr	30-39 yr	40-49 yr	50-59 yr	60-69 yr	70-79 yr	80-89 yr
10-19 yr	0.00								
20-29 yr	0.00	0.00							
30-39 yr	0.00	0.00	0.00						
40-49 yr	0.00	0.00	0.00	0.00					
50-59 yr	0.17	0.17	0.17	0.17	0.17				
60-69 yr	1.04	1.04	1.04	1.04	1.04	0.87			
70-79 yr	3.41	3.41	3.41	3.41	3.41	3.24	2.36		
80-89 yr	15.42 ****	15.42 ****	15.42 ****	15.42 ****	15.42 ****	15.25 ****	14.38 ****	12.01 ****	
90+ yr	36.60 ****	36.60 ****	36.60 ****	36.60 ****	36.60 ****	36.44 ****	35.56 ****	33.20 ****	21.19****

Table S6. Tukey's post-hoc tests of pairwise age-group comparisons in COVID-19 case fatality rates within each of nine countries. Values in Table are Q-stat. P values are indicated by * for <0.05, ** for <0.01, *** for <0.001, and **** for <0.0001.

Italy

	0-9 yr	10-19 yr	20-29 yr	30-39 yr	40-49 yr	50-59 yr	60-69 yr	70-79 yr	80-89 yr
10-19 yr	0.01								
20-29 yr	0.00	0.01							
30-39 yr	0.01	0.02	0.013						
40-49 yr	0.08	0.09	0.084	0.07					
50-59 yr	0.26	0.28	0.27	0.25	0.18				
60-69 yr	1.04	1.06	1.05	1.03	0.96	0.78			
70-79 yr	3.21	3.22	3.21	3.20	3.13	2.95	2.17		
80-89 yr	7.41****	7.43****	7.42****	7.40****	7.33****	7.15****	6.37***	4.20	
90+ yr	10.59****	10.61****	10.60****	10.58****	10.51****	10.33****	9.55****	7.38****	3.18

UK

	0-4 yr	5-9 yr	10-19 yr	20-29 yr	30-39 yr	40-49 yr	50-59 yr	60-69 yr	70-79 yr
5-9 yr	0.14								
10-19 yr	0.23	0.30							
20-29 yr	0.09	0.38	0.08						
30-39 yr	0.47	0.25	0.05	0.13					
40-49 yr	0.16	0.31	0.61	0.69	0.56				
50-59 yr	1.71	1.56	1.86	1.94	1.81	1.25			
60-69 yr	6.33***	6.17***	6.47***	6.55***	6.42***	5.86**	4.61*		
70-79 yr	19.33****	19.17****	19.47****	19.55****	19.42****	18.86****	17.61****	13.00****	
80+ yr	35.01****	34.86****	35.16****	35.24****	35.10****	34.54****	33.30****	28.69****	15.68****

South Korea

	0-9 yr	10-19 yr	20-29 yr	30-39 yr	40-49 yr	50-59 yr	60-69 yr	70-79 yr
10-19 yr	0.00							
20-29 yr	0.00	0.00						
30-39 yr	0.05	0.05	0.05					
40-49 yr	0.06	0.06	0.06	0.01				

50-59 yr	0.15	0.15	0.15	0.10	0.09			
60-69 yr	1.54	1.54	1.54	1.49	1.48	1.39		
70-79 yr	5.11*	5.11*	5.11*	5.07*	5.06*	4.97*	3.58	
80+ yr	10.13****	10.13****	10.13****	10.09****	10.06****	9.99****	8.60****	5.02*

Germany

	0-4 yr	5-14 yr	15-34 yr	35-59 yr	60-79 yr
5-14 yr	0.02				
15-34 yr	0.00	0.02			
35-59 yr	0.43	0.44	0.43		
60-79 yr	4.94**	4.95**	4.94**	4.51*	
80+ yr	22.76****	22.78****	22.76****	22.34****	17.83****

South Africa

	0-9 yr	10-19 yr	20-29 yr	30-39 yr	40-49 yr	50-59 yr	60-69 yr	70-79 yr
10-19 yr	0.21							
20-29 yr	0.04	0.17						
30-39 yr	0.29	0.50	0.34					
40-49 yr	1.24	1.46	1.29	0.95				
50-59 yr	3.76	3.97	3.81	3.47	2.52			
60-69 yr	9.43****	9.64****	9.48****	9.14****	8.19****	5.67**		
70-79 yr	14.56****	14.78****	14.61****	14.27****	13.32****	10.80****	5.13**	
80+ yr	16.94****	17.15****	16.98****	16.65****	15.69****	13.18****	7.51****	2.37

Portugal

	0-9 yr	10-19 yr	20-29 yr	30-39 yr	40-49 yr	50-59 yr	60-69 yr	70-79 yr
10-19 yr	0.00							
20-29 yr	0.03	0.03						
30-39 yr	0.05	0.05	0.02					
40-49 yr	0.20	0.20	0.17	0.15				
50-59 yr	0.73	0.73	0.70	0.68	0.53			
60-69 yr	3.18	3.18	3.15	3.13	2.97	2.45		

70-79 yr	10.22****	10.22****	10.19****	10.17****	10.02****	9.49****	7.04****	
80+ yr	24.20****	24.20****	24.17****	24.15****	24.00****	23.47****	21.02****	13.98****

Turkey

	0-14 yr	15-24 yr	25-49 yr	50-64 yr	65-79 yr
15-24 yr	0.02				
25-49 yr	0.10	0.12			
50-64 yr	1.64	1.66	1.54		
65-79 yr	8.29****	8.31****	8.19****	6.65***	
80+ yr	20.46****	20.48****	20.37****	18.82****	12.17****

Peru

	0-9 yr	10-19 yr	20-29 yr	30-39 yr	40-49 yr	50-59 yr	60-69 yr	70-79 yr
10-19 yr	0.18							
20-29 yr	0.34	0.52						
30-39 yr	0.28	0.10	0.62					
40-49 yr	1.99	1.81	2.33	1.71				
50-59 yr	5.91**	5.73**	6.25***	5.62**	3.92			
60-69 yr	15.94****	15.76****	16.28****	15.65****	13.95****	10.03****		
70-79 yr	28.82****	28.64****	29.16****	28.54****	26.83****	22.91****	12.88****	
80+ yr	45.60****	45.42****	45.94****	45.32****	43.61****	39.70****	29.66****	16.78****

Netherlands

	0-9 yr	10-19 yr	20-29 yr	30-39 yr	40-49 yr	50-59 yr	60-69 yr	70-79 yr	80-89 yr
10-19 yr	0.00								
20-29 yr	0.00	0.00							
30-39 yr	0.00	0.00	0.00						
40-49 yr	0.00	0.00	0.00	0.00					
50-59 yr	0.31	0.31	0.31	0.31	0.31				
60-69 yr	1.63	1.63	1.63	1.63	1.63	1.32			
70-79 yr	6.62***	6.62***	6.62***	6.62***	6.62***	6.31***	5.00*		
80-89 yr	14.71****	14.71****	14.71****	14.71****	14.71****	14.40****	13.08****	8.08****	

90+ yr	21.30****	21.30****	21.30****	21.30****	21.30****	20.99****	19.67****	14.68****	6.60***

Table S7. Tukey's post-hoc tests of pairwise age-group comparisons in the weekly changes of case proportions caused by COVID-19 in one country that showed significant differences between at least two age groups. Values in Table are Q-stat. P values are indicated by * for <0.05, ** for <0.01, *** for <0.001, and **** for <0.0001.

UK

	0-4 yr	5-9 yr	10-19 yr	20-29 yr	30-39 yr	40-49 yr	50-59 yr	60-69 yr	70-79 yr
5-9 yr	0.09								
10-19 yr	1.24	1.33							
20-29 yr	1.33	1.42	0.09						
30-39 yr	0.51	0.60	0.73	0.82					
40-49 yr	0.24	0.32	1.01	1.09	0.28				
50-59 yr	0.06	0.15	1.18	1.27	0.45	0.18			
60-69 yr	0.32	0.23	1.56	1.65	0.83	0.56	0.38		
70-79 yr	1.14	1.05	2.38	2.47	1.65	1.37	1.19	0.82	
80+ yr	5.44**	5.35**	6.68***	6.77***	5.95**	5.68**	5.50**	5.12*	4.30

Table S8. ANOVA and Tukey's results performed on logit transformed case data compared to relative proportion data. Logit transformation was performed on Italy, US, and UK data, which showed the largest number of significant correlations.

F values based on logit data			
Country	Age-group	Sex	Number of data points
Italy	5.20****	0.02 NS	39 (dropped two weeks because of NA logit values)
US	27.48****	0.17 NS	42
UK	20.84****	1.80 NS	21

F values based on relative proportions data			
Country	Age-group	Sex	Number of data points
Italy	39.59****	1.64 NS	41
US	186.23****	6.91**	42
UK	36.70****	5.77*	21

Italy Logit Tukey's Post-Hoc Test									
	0-9 years	10-19 years	20-29 years	30-39 years	40-49 years	50-59 years	60-69 years	70-79 years	80-89 years
10-19 years	2.49 NS								
20-29 years	5.96**	3.46 NS							
30-39 years	5.44**	2.94 NS	0.52 NS						
40-49 years	5.20*	2.71 NS	0.76 NS	0.23 NS					
50-59 years	5.38**	2.89 NS	0.57 NS	0.05 NS	0.18 NS				
60-69 years	4.25 NS	1.76 NS	1.70 NS	1.18 NS	0.95 NS	1.13 NS			
70-79 years	4.18 NS	1.69 NS	1.77 NS	1.25 NS	1.02 NS	1.20 NS	0.07 NS		
80-89 years	6.12***	3.62 NS	0.16 NS	0.68 NS	0.92 NS	0.73 NS	1.86 NS	1.93 NS	
90+ years	8.58****	6.08***	2.62 NS	3.14 NS	3.37 NS	3.19 NS	4.32 NS	4.39 NS	2.46 NS

Italy Relative Proportions Tukey's Post-Hoc Test

	0-9 years	10-19 years	20-29 years	30-39 years	40-49 years	50-59 years	60-69 years	70-79 years	80-89 years
10-19 years	4.31****								
20-29 years	11.70****	7.39****							
30-39 years	8.49****	4.19	3.21						
40-49 years	6.95****	2.64	4.76*	1.55					
50-59 years	7.15*	2.84	4.55*	1.35	0.22				
60-69 years	4.69*	0.38	7.01****	3.81	2.26	2.46			
70-79 years	4.56****	0.25	7.15****	3.94	2.39	2.59	0.13		
80-89 years	10.58****	6.27***	1.121	2.09	3.64	3.44	5.90**	6.03***	
90+ years	23.29****	18.98****	11.58****	14.79****	16.34****	16.14****	19.60****	18.73****	12.70****

US Logit Tukey's Post-Hoc Test								
	0-9 years	10-19 years	20-29 years	30-39 years	40-49 years	50-59 years	60-69 years	70-79 years
10-19 years	8.94****							
20-29 years	16.17****	7.23****						
30-39 years	15.57****	6.63***	0.60 NS					
40-49 years	15.94****	7.00****	0.23 NS	0.37 NS				
50-59 years	15.38****	6.44***	0.79 NS	0.19 NS	0.56 NS			
60-69 years	13.02****	4.09 NS	3.15 NS	2.55 NS	2.92 NS	2.36 NS		
70-79 years	12.01****	3.07 NS	4.16 NS	3.56 NS	3.93 NS	3.37 NS	1.01 NS	
80-89 years	15.20****	6.26***	0.97 NS	0.37 NS	0.74 NS	0.18 NS	2.17 NS	3.19 NS

US Relative Proportions Tukey's Post-Hoc Test								
	0-9 years	10-19 years	20-29 years	30-39 years	40-49 years	50-59 years	60-69 years	70-79 years
10-19 years	17.04****							
20-29 years	41.71****	24.67****						
30-39 years	37.21****	20.17****	4.5*					
40-49 years	39.03****	22****	2.67	1.83				

50-59 years	36.29****	19.25****	5.42**	0.92	2.75			
60-69 years	26.25****	9.21****	15.46****	10.95****	12.78****	10.03****		
70-79 years	22.66****	5.62**	19.05****	14.55****	16.38****	13.63****	3.6	
80-89 years	37.74****	20.7****	3.97	0.53	1.29	1.45	11.49****	15.08****

UK Logit Tukey's Post-Hoc Test									
	0-4 years	5-9 years	10-19 years	20-29 years	30-39 years	40-49 years	50-59 years	60-69 years	70-79 years
5-9 years	0.25 NS								
10-19 years	5.51**	5.26**							
20-29 years	12.17****	11.92****	6.66***						
30-39 years	10.92****	10.67****	5.41**	1.25 NS					
40-49 years	10.24****	9.99****	4.73*	1.93 NS	0.68 NS				
50-59 years	9.19****	8.94****	3.68 NS	2.98 NS	1.73 NS	1.05 NS			
60-69 years	7.02****	6.77***	1.51 NS	5.15*	3.90 NS	3.22 NS	2.17 NS		
70-79 years	5.54**	5.28**	0.03 NS	6.63***	5.38**	4.70**	3.66 NS	1.48 NS	
80+ years	12.70****	12.44****	7.19****	0.53 NS	1.78 NS	2.46 NS	3.51 NS	5.68**	7.16****

UK Relative Proportions Tukey's Post-Hoc Test									
	0-4 years	5-9 years	10-19 years	20-29 years	30-39 years	40-49 years	50-59 years	60-69 years	70-79 years
5-9 years	0.01								
10-19 years	4.70*	4.69*							
20-29 years	14.21****	14.20****	9.51****						
30-39 years	10.45****	10.45****	5.75**	3.755					
40-49 years	8.92****	8.91****	4.22	5.287**	1.53				
50-59 years	7.10****	7.09****	2.4	7.113****	3.36	1.83			
60-69 years	4.11	4.11	0.59	10.095****	6.34***	4.81*	2.98		

70-79 years	3.25	3.24	1.45	10.961****	7.21****	5.67**	3.85	0.87	
80+ years	18.72****	18.71****	14.02****	4.512****	8.27****	9.8****	11.63****	14.61****	15.47****

Table S9. Temporal patterns of COVID-19 infection cases using the formula of N_{t+1}/N_t between weeks

P values are indicated by * for <0.05/n, ** for <0.01/n, *** for <0.001/n, and **** for <0.0001/n, where n is the number of age groups for that specific country

Italy Cases

	0-9 yr	10-19 yr	20-29 yr	30-39 yr	40-49 yr	50-59 yr	60-69 yr	70-79 yr	80-89 yr	90+ yr
Male	0.17NS	0.08NS	0.08NS	0.12NS	0.24NS	0.20NS	0.25NS	0.30NS	0.27NS	0.18NS
Female	0.13NS	0.03NS	0.09NS	0.18NS	0.20NS	0.19NS	0.22NS	0.23NS	0.19NS	0.17NS

UK Cases

	0-4 yr	5-9 yr	10-19 yr	20-29 yr	30-39 yr	40-49 yr	50-59 yr	60-69 yr	70-79 yr	80+ yr
Male	0.11NS	-0.26NS	-0.25NSS	-0.26NS	-0.26NS	-0.25NS	-0.24NS	-0.17NS	0.19NS	0.17NS
Female	0.11NS	-0.27NS	-0.27NS	-0.24NS	-0.24NS	-0.24NS	-0.24NS	-0.21NS	0.07NS	-0.00NS

USA Cases

	0-9 yr	10-19 yr	20-29 yr	30-39 yr	40-49 yr	50-59 yr	60-69 yr	70-79 yr	80-89 yr
Male	-0.44*	-0.38NS	-0.38NSS	-0.34NS	-0.34NS	-0.33NS	-0.31NS	-0.32NS	-0.36NS
Female	-0.44*	-0.45*	-0.39NS	-0.36NS	-0.35NS	-0.34NS	-0.32NS	-0.34NS	-0.39NS
